# Trend of fetal echocardiography use and comparison of medical costs for congenital heart disease treatment based on fetal echocardiography use in a Korean single center

**DOI:** 10.3389/fped.2023.933623

**Published:** 2023-06-30

**Authors:** Ho-Jung Choi, Mi-Kyoung Song, Sang-Yun Lee, Gi-Beom Kim, Eun-Jung Bae, Joong-Shin Park, Jong-Kwan Jun, Hye-Won Kwon, Hong-Gook Lim, Woong-Han Kim

**Affiliations:** ^1^ Department of Pediatrics, Seoul National University Hospital, Seoul, Republic of Korea; ^2^ Department of Obstetrics and Gynecology, Seoul National University Hospital, Seoul, Republic of Korea; ^3^ Department of Thoracic and Cardiovascular Surgery, Seoul National University Hospital, Seoul, Republic of Korea

**Keywords:** fetal echocardiography, prenatal diagnosis, congenital heart disease, hospitalization duration, medical cost

## Abstract

**Background:**

The rate of the prenatal diagnosis of congenital heart disease is increasing along with advances in fetal echocardiography techniques. Here, we aimed to investigate the trend of the use of fetal echocardiography over time and to compare the medical costs of congenital heart disease treatment according to whether fetal echocardiography was performed.

**Methods:**

We reviewed our hospital’s database, and patients who underwent the first surgery for congenital heart disease within 30 days of birth during 2005–2007, 2011–2013, and 2017–2019 were included. The severity of congenital heart disease diagnosed in each case was evaluated according to The Society of Thoracic Surgeons-European Association for Cardio-Thoracic Surgery Congenital Heart Surgery Mortality Scores (STS-EACTS Mortality Scores) and Mortality Categories (STAT Mortality Categories).

**Results:**

In total, 375 patients were analyzed, and fetal echocardiography use increased significantly after the 2010s compared with in 2005–2007 (19.1% vs. 39%, *p *= 0.032 in Mortality Category 1–3; 15.5% vs. 69.5%, *p *= 0.000 in Mortality Category 4–5). Additionally, the mean STS-EACTS Mortality Score was higher in prenatally diagnosed patients than in postnatally diagnosed patients (2.287 vs. 1.787, *p *= 0.001). In the recent period, there was no significant difference in hospitalization durations and medical costs according to whether or not fetal echocardiography was performed.

**Conclusions:**

This single center study showed the use of fetal echocardiography is increasing. Further, prenatal diagnosis with fetal echocardiography causing no differences in medical costs in recent years. Therefore, we suggest that fetal echocardiography can be applied more widely without increasing the economic burden.

## 1. Introduction

The rate of the prenatal diagnosis of congenital heart disease (CHD) is increasing owing to the advances in fetal echocardiography techniques. Fetal echocardiography enables prenatal diagnosis, which allows for immediate disease management following birth as well as for appropriate parent counseling (1, 2). A meta-analysis revealed that this modality has a sensitivity of 68.5% and specificity of 99.8% (3). Additionally, prenatal diagnosis is expected to contribute to improving the outcomes in CHD. In infants with CHD, which is dependent on the patency of the ductus arteriosus, it is critical to maintain ductus blood flow for stable hemodynamics. The early use of prostaglandin-E1 results in better preoperative conditions, and prenatal detection with fetal echocardiography can prevent unexpected emergency conditions (2, 4).

In the early 2000s, a fetal echocardiography clinic commenced operations in our center, and currently, more than 200 patients are being diagnosed with CHD every year. Although there are several studies on fetal echocardiography in other countries, data regarding this modality are sparse in South Korea. Further, the impact of fetal echocardiography on medical expenses has not been evaluated in CHD. Considering the high cost of CHD treatment, it is important that medical costs do not increase significantly. Here, we aimed to review the trend in the use of fetal echocardiography over time and its impact on the medical expenses associated with CHD in neonates who received cardiovascular surgery in our center. The hospitalization duration and medical costs were evaluated, with the aim of identifying whether early diagnosis with fetal echocardiography increased the economic burden.

## 2. Materials and methods

### 2.1. Study population

We reviewed clinical data retrieved from the Seoul National University Hospital Patients Research Environment system and database of cardiovascular surgery for CHD, and selected patients who underwent their first surgery within 30 days of birth. Patients who underwent closure of persistent ductus arteriosus during preterm infancy were excluded. To evaluate the temporal trend of the use of fetal echocardiography and associated medical expenses, we collected data from three different periods: 2005–2007, 2011–2013, and 2017–2019. Clinical data, such as gestational age (GA), birth weight, birth height (if available), sex, diagnosis, surgery date and procedure, age at surgery, the presence or absence of fetal echocardiography results, total hospitalization duration including duration of intensive care unit (ICU) stay and duration of postoperative stay, and total medical costs, were investigated.

This study was conducted after obtaining approval from the Institutional Review Board at our institution (H-2005-205-1127, approval date: June 15, 2020). The requirement for informed consent was waived because of the retrospective study design.

### 2.2. Fetal echocardiography

Fetal echocardiography was performed on request when abnormal findings were suspected in obstetrics department. It was done by a pediatric cardiologist who worked in the division of cardiology for more than 5 years. And it was usually performed once in early 20 weeks of pregnancy or 30 weeks of pregnancy or at both times.

Ultrasound systems used for fetal echocardiography had capabilities for performing 2-dimensional, M-mode, and Doppler imaging. Frames rates were 80 to 100 Hz frequency because the heart rate of fetus is over 140 beats per minute. Phased array transducers with fundamental frequencies between 4 and 12 MHz are generally used.

The essential components of the fetal echocardiogram were made up of anatomic overview, cardiac imaging views/sweeps, doppler examination, measurement data, and examination of rhythm and rate (1).

### 2.3. Duration of hospitalization and total medical costs

The duration of hospitalization was evaluated in terms of three aspects: total hospitalization duration, ICU stay duration, and postoperative stay duration. Total medical costs were set as the hospitalization expenses for the first operation. To calculate the total medical cost, 1,000 South Korean won (￦) was converted to 1 United States dollar ($), and the total was rounded off to the nearest dollar. Considering the inflation, change of operational costs and techniques, the comparison was done within each period.

### 2.4. Disease severity

Considering the impact of the severity of CHD and risk of surgery on economic burden, each patient was classified based on The Society of Thoracic Surgeons—European Association for Cardio-Thoracic Surgery Congenital Heart Surgery Mortality Scores (STS-EACTS Mortality Scores) and Mortality Categories (STAT Mortality Categories) (5). A higher Mortality Score and Mortality Category indicated higher mortality associated with congenital heart surgery, which indicated a diagnosis of more severe CHD. Patients were divided into two groups with Mortality Category 1–3 and 4–5, and comparisons were performed. We grouped the Mortality category into low-risk group (1–3) and high-risk group (4,5) based on the previous studies that reported significant difference in mortality between two groups (6, 7).

### 2.5. Statistical analysis

We used Statistical Package for the Social Sciences (SPSS), version 26 (SPSS Inc., IBM, Chicago, IL, USA), for the statistical analysis of data. To compare the fetal echocardiography usage between the 3 different time periods, we used one way analysis of variance (ANOVA). A chi-square test or Fisher’s exact test was used to compare categorical variables, and a Mann-Whitney test was used to compare the hospitalization periods and costs. Results were considered statistically significant when the *p* value was <0.05.

## 3. Results

### 3.1. Patient characteristics

**Figure 1:**
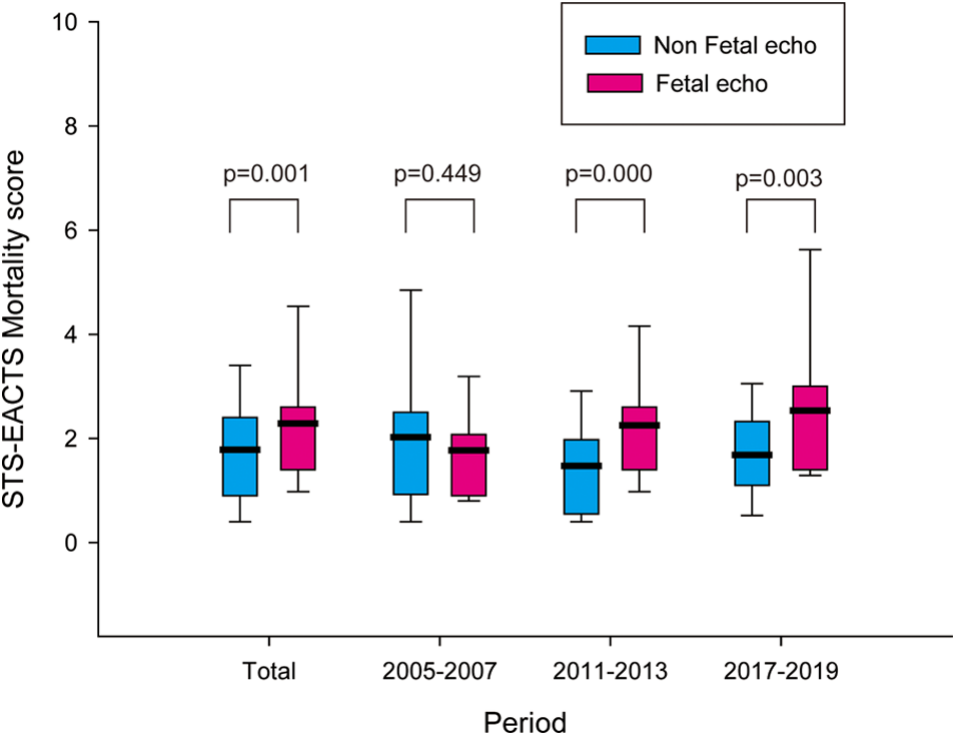
STS-EACTS mortality score in the non-fetal echocardiography and fetal echocardiography groups in 2005−2007, 2011−2013, 2017−2019, and the total study period. The bold line indicates the mean value. STS, Society of Thoracic Surgeons; EACTS, European Association for Cardio-Thoracic Surgery.

The baseline characteristics of the reviewed patients are shown in Table 1. A total of 375 patients were included. Among them, 126, 159, and 90 patients were included for the periods of 2005–2007, 2011–2013, and 2017–2019, respectively. There were 42 (11.2%) preterm patients, and the median birth weight was 3.15 kg (range, 1.23–5.2 kg). There were 187 patients with STAT Mortality Category 1–3 and 188 patients with STAT Mortality Category 4–5, indicative of more severe CHD. Through the entire study period, the mean STS-EACTS Mortality Score in the non-fetal echocardiography group was 1.787 (±1.3031), whereas that in the fetal echocardiography group was 2.287 (±1.4972) (*p = 0.001*) (Figure 1). There was no significant difference in the mean Mortality Score in 2005–2007 (2.026 vs. 1.773, *p *= 0.449); however, in 2011–2013 and 2017–2019, the mean Mortality Score was significantly higher in the prenatally diagnosed group (1.471 vs. 2.252, *p *= 0.000 and 1.681 vs. 2.536, *p *= 0.003, respectively).

**Table 1:** Baseline characteristics of the patients by time period.

Variables	2005–2007 (*n* = 126)	2011–2013 (*n* = 159)	2017–2019 (*n* = 90)	*p* value
Gestational age (weeks)				*0*.*938*
<30 + 0 wk	1 (0.8)	1 (0.6)	0 (0)	* *
30 + 0∼33 + 6 wk	3 (2.4)	2 (1.3)	2 (2.2)	* *
34 + 0∼36 + 6 wk	10 (7.9)	15 (9.4)	8 (8.9)	* *
37 + 0∼39 + 6 wk	78 (61.9)	107 (67.3)	56 (62.2)	* *
40 + 0 wk ≤	34 (27.0)	34 (21.4)	24 (26.7)	* *
Birth weight (kg)				*0*.*475*
<1.5 kg	2 (1.6)	2 (1.3)	1 (1.1)	* *
1.5∼2.5 kg	8 (6.3)	20 (12.6)	13 (14.4)	* *
2.5 kg <	116 (92.1)	137 (86.2)	76 (84.4)	* *
Sex				*0*.*806*
Male	76 (60.3)	100 (62.9)	53 (58.9)	* *
Female	50 (39.7)	59 (37.1)	37 (41.1)	* *
STS-EACTS Mortality Score	1.982 (±1.4177)	1.898 (±1.3392)	2.232 (±1.5225)	*0*.*195*
STAT Mortality Category				*0*.*088*
1	7 (5.7)	10 (6.3)	1 (1.1)	* *
2	30 (23.9)	34 (21.4)	15 (16.7)	* *
3	31 (24.7)	33 (20.7)	26 (28.9)	* *
4	54 (43.0)	82 (51.6)	44 (48.9)	* *
5	4 (3.3)	0 (0)	4 (4.4)	* *
Fetal echocardiography				*0*.*000*
No	104 (82.5)	72 (45.3)	32 (35.6)	* *
Yes	22 (17.5)	87 (54.7)	58 (64.4)	* *
Operation type				*0.167*
Total correction	93 (73.8)	105 (66.0)	56 (62.2)	* *
Palliative, stage operation	33 (26.2)	54 (34.0)	34 (37.8)	* *

Values are presented as *n* (%) apart from the STS-EACTS Mortality Score, which is presented as mean (±SD).

STS, Society of Thoracic Surgeons; EACTS, European Association for Cardio-Thoracic Surgery; STAT, The Society of Thoracic Surgeons-European Association for Cardio-Thoracic Surgery; SD, standard deviation.

### 3.2. Fetal echocardiography use

Of all the registered patients, 167 patients (44.5%) underwent fetal echocardiography due to a suspicion of CHD. Among this, the prenatal diagnosis was consistent with the actual diagnosis in 89.8% of patients (*n* = 150).

**Figure 2:**
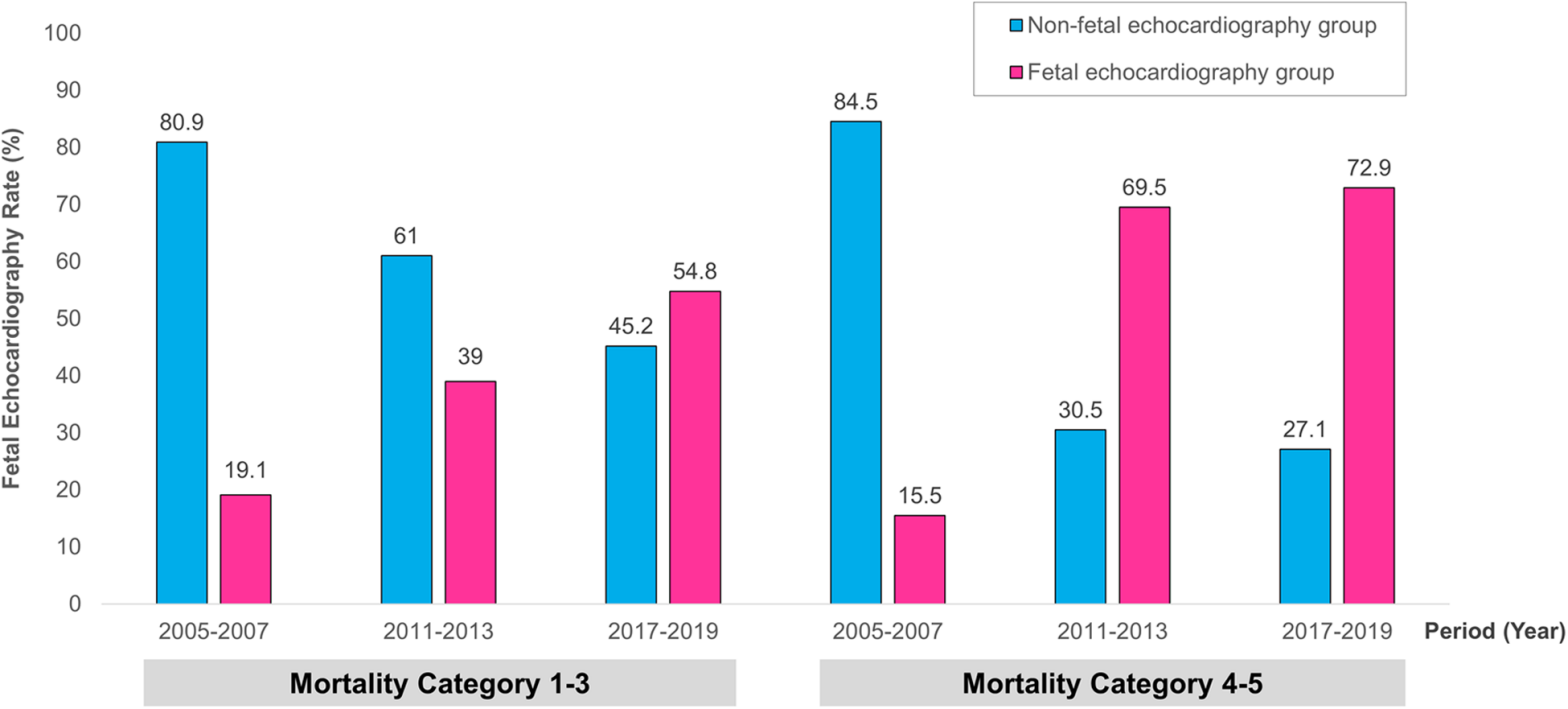
The trend of fetal echocardiography use according to the time period. This was compared between the two groups with STAT Mortality Category 1−3 and 4−5, respectively. STAT, The Society of Thoracic Surgeons-European Association for Cardio-Thoracic Surgery.

We further analyzed the trend of fetal echocardiography use over time according to the Mortality Category (Figure 2). Between the periods of 2005–2007 and 2011–2013, the fetal echocardiography usage ratio increased from 19.1% to 39% in Mortality Category 1–3 (*p *= 0.032) and from 15.5% to 69.5% in Mortality Category 4–5 (*p *= 0.000). And in the recent periods of 2017–2019, fetal echocardiography usage of Mortality Category 1–3 and 4–5 were 54.8% and 72.9%, respectively.

### 3.3. Hospitalization duration and medical costs

The total duration of hospitalization, duration of ICU stay, duration of postoperative stay, and total medical cost according to whether or not fetal echocardiography was performed are shown in Table 2. In the past period of 2005–2007, fetal echocardiography group of Mortality Category 4–5 showed longer total hospitalization period and ICU stay with increased medical costs. In 2011–2013, only total costs for Mortality Category 1–3 was higher in fetal echocardiography group. In the most recent period of 2017–2019, there was no significant difference between the non-fetal echocardiography and fetal echocardiography group.

**Table 2:** Total duration of hospitalization, duration of ICU stay, duration of postoperative stay, and total medical costs according to the STAT mortality category and application of fetal echocardiography.

2005–2007 (*n* = 126)						
	Mortality Category 1–3 (*n* = 68)			Mortality Category 4–5 (*n* = 58)		
Fetal echocardiography	No (*n* = 55)	Yes (*n* = 13)	*p* value	No (*n* = 49)	Yes (*n* = 9)	*p* value
Total hospitalization (days)	19 (13–31)	26 (19–28.5)	*0*.*117*	21 (14–31)	30 (23.5–65.5)	*0*.*041*
ICU stay (days))	11 (6–18)	17 (14–26.5)	*0*.*014*	11 (7.5–20)	30 (15.5–49.5)	*0*.*019*
Postoperative stay (days)	13 (9–21)	19 (11–22.5)	*0*.*268*	13 (9.5–27.5)	19 (13.5–44.0)	*0*.*246*
Total cost ($)	16,106 (12,356–21,308)	18,763 (17,516–23,319)	*0*.*072*	17,187 (13,144–22,248)	22,412 (19,423–33,751)	*0*.*040*
2011–2013 (*n* = 159)						
	Mortality Category 1–3 (*n* = 77)			Mortality Category 4–5 (*n* = 82)		
Fetal echocardiography	No (*n* = 47)	Yes (*n* = 30)	*p* value	No (*n* = 25)	Yes (*n* = 57)	*p* value
Total hospitalization (days)	20 (13–28)	22.5 (16–27)	*0*.*487*	25 (21–39)	28 (20–43.5)	*0*.*817*
ICU stay (days)	11 (6–22)	16 (8–24.25)	*0*.*177*	19 (12.5–27)	24 (15.5–32.5)	*0*.*226*
Postoperative stay (days)	13 (9–20)	14 (10.75–19)	*0*.*691*	18 (13–30)	17 (11–32)	*0*.*583*
Total cost ($)	24,520 (18,650–32,324)	29,896 (25,535–34,691)	*0*.*025*	34,909 (25,237–46,049)	35,432 (27,086–46,367)	*0*.*569*
2017–2019 (*n* = 90)						
	Mortality Category 1–3 (*n* = 42)			Mortality Category 4–5 (*n* = 48)		
Fetal echocardiography	No (*n* = 19)	Yes (*n* = 23)	*p* value	No (*n* = 13)	Yes (*n* = 35)	*p* value
Total hospitalization (days)	22 (17–32)	27 (19–29)	*0*.*362*	33 (15.5–74.5)	33 (23–55)	*0*.*693*
ICU stay (days)	17 (8–25)	18 (14–22)	*0*.*440*	28 (9–62.5)	26 (15–50)	*0*.*585*
Postoperative stay (days)	14 (9–25)	13 (10–25)	*0*.*820*	14 (12–64.5)	16 (12–37)	*0*.*710*
Total cost ($)	48,712 (43,685–57,497)	57,860 (47,464–73,312)	*0*.*071*	54,224 (39,906–110,769)	55,966 (49,595–96,757)	*0*.*862*

Duration of hospitalization and total cost are presented as median [interquartile range (IQR)].

CHD, congenital heart disease; ICU, intensive care unit; STAT, The Society of Thoracic Surgeons-European Association for Cardio-Thoracic Surgery; SD, standard deviation.

## 4. Discussion

Despite the increasing interest in fetal echocardiography, there are few studies addressing the recent trend of its use in South Korea. Our analysis of patients who underwent surgical repair within 30 days of birth revealed that the rate of the prenatal diagnosis of CHD through fetal echocardiography has been increasing significantly over time, especially between 2005 and 2007 and 2011–2013. This result implies that fetal echocardiography came into widespread use since the 2010s in South Korea, which may be due to advances in obstetrical screening and fetal echocardiography techniques.

Subsequently, we analyzed whether prenatal diagnosis with fetal echocardiography results in an increase in the hospitalization duration or total medical costs. In the past period of 2005–2007, there was some increased hospitalization period or medical cost in fetal echocardiography group, especially in high-risk diagnosis group. But as time goes by, only the increase of medical cost was shown for the low-risk group in 2011–2013, and there was no difference in hospitalization period and total cost between the prenatally diagnosed group and postnatally diagnosed group in 2017–2019. Our results revealed that prenatal diagnosis with fetal echocardiography did not lead to significant differences in the hospitalization duration or medical costs in patients with CHD in both the Mortality Score groups in recent period. Patients with prenatally detected CHD are directly admitted to the neonatal ICU and are provided preoperative management through procedures, such as a continuous infusion of prostaglandin-E1 or several follow-up examinations. Therefore, it can be considered that the medical expenses would be higher in the prenatally diagnosed group. Considering this, it is notable that prenatal diagnosis with fetal echocardiography can be applied more widely without increasing the economic burden as early diagnosis is expected to further stabilize the preoperative conditions. These results can be attributed to the substantial improvement in surgical techniques and postoperative care for CHD over time. Although it is still the leading cause of infant death, the mortality associated with CHD has decreased dramatically over the decades through these improvements (8). In our analysis, the cost regarding prenatal diagnosis is not included in the analyzed medical costs of fetal echocardiography group. But unanalyzed expenses of the other group, such as emergency transportation costs or medical treatment costs before admission, should also be considered (9). Postnatally diagnosed patients usually undergo several examinations, including echocardiography, at other hospitals, and some of them are transported to our hospital for emergency surgery. Thus, prenatal diagnosis can reduce overlapping examinations, thereby resulting in economic advantages. Meanwhile, such prenatal examinations are expected to intensify the centralization of CHD patients in tertiary hospitals.

The postoperative outcomes were not evaluated in this study. For us to perform this evaluation, data from cases involving mortality before the transfer to our hospital and surgeries at regional hospitals should be included; this is a limitation associated with a single hospital study.

In conclusion, this is the first study to evaluate the trend and medical cost of fetal echocardiography in the Republic of Korea. There have been developments in fetal echocardiography techniques over time, and the ratio of the application of this diagnostic modality has significantly increased since the 2010s. Additionally, prenatal diagnosis with fetal echocardiography did not increase the medical expenses in recent years. Therefore, we suggest that fetal echocardiography can be applied more widely without increasing the economic burden and that further studies that include the clinical outcomes would be helpful in gaining more insights regarding its diagnostic value.

## Data Availability

The raw data supporting the conclusions of this article will be made available by the authors, without undue reservation.

## References

[1] Rychik J Ayres N Cuneo B Gotteiner N Hornberger L Spevak PJ American Society of echocardiography guidelines and standards for performance of the fetal echocardiogram. J Am Soc Echocardiogr. (2004) 17:803–10. 10.1016/j.echo.2004.04.011 15220910

[2] Sharland G. Fetal cardiac screening and variation in prenatal detection rates of congenital heart disease: why bother with screening at all? Future Cardiol. (2012) 8:189–202. 10.2217/fca.12.15 22413979

[3] Zhang YF Zeng XL Zhao EF Lu HW. Diagnostic value of fetal echocardiography for congenital heart disease: a systematic review and meta-analysis. Medicine (Baltimore). (2015) 94:e1759. 10.1097/MD.0000000000001759 26496297 PMC4620824

[4] Singh Y Mikrou P. Use of prostaglandins in duct-dependent congenital heart conditions. Arch Dis Child Educ Pract Ed. (2018) 103:137–40. 10.1136/archdischild-2017-313654 29162633

[5] Barach P Jacobs JP Lipshultz SE Laussen PC. Pediatric and congenital cardiac care. London: Springer (2015).

[6] Pasquali SK Gaies M Banerjee M Zhang W Donohue J Russell M The quest for precision medicine: unmeasured patient factors and mortality after congenital heart surgery. Ann Thorac Surg. (2019) 108:1889–94. 10.1016/j.athoracsur.2019.06.031 31398358 PMC7075483

[7] Pasquali SK Thibault D O’Brien SM Jacobs JP Gaynor JW Romano JC National variation in congenital heart surgery outcomes. Circulation. (2020) 142:1351–60. 10.1161/CIRCULATIONAHA.120.046962 33017214 PMC7539149

[8] Wu W He J Shao X. Incidence and mortality trend of congenital heart disease at the global, regional, and national level, 1990–2017. Medicine (Baltimore). (2020) 99:e20593. 10.1097/MD.0000000000020593 32502030 PMC7306355

[9] Jegatheeswaran A Oliveira C Batsos C Moon-Grady AJ Silverman NH Hornberger LK Costs of prenatal detection of congenital heart disease. Am J Cardiol. (2011) 108:1808–14. 10.1016/j.amjcard.2011.07.052 21907953

